# Global Misinformation Spillovers in the Vaccination Debate Before and During the COVID-19 Pandemic: Multilingual Twitter Study

**DOI:** 10.2196/44714

**Published:** 2023-05-24

**Authors:** Jacopo Lenti, Yelena Mejova, Kyriaki Kalimeri, André Panisson, Daniela Paolotti, Michele Tizzani, Michele Starnini

**Affiliations:** 1 Department of Computer, Control, and Management Engineering Antonio Ruberti Sapienza University of Rome Rome Italy; 2 CENTAI Institute S.p.A. Turin Italy; 3 Institute for Scientific Interchange Foundation Turin Italy; 4 Departament de Fisica, Universitat Politecnica de Catalunya Barcelona Spain

**Keywords:** vaccination hesitancy, vaccine, misinformation, Twitter, social media, COVID-19

## Abstract

**Background:**

Antivaccination views pervade online social media, fueling distrust in scientific expertise and increasing the number of vaccine-hesitant individuals. Although previous studies focused on specific countries, the COVID-19 pandemic has brought the vaccination discourse worldwide, underpinning the need to tackle low-credible information flows on a global scale to design effective countermeasures.

**Objective:**

This study aimed to quantify cross-border misinformation flows among users exposed to antivaccination (no-vax) content and the effects of content moderation on vaccine-related misinformation.

**Methods:**

We collected 316 million vaccine-related Twitter (Twitter, Inc) messages in 18 languages from October 2019 to March 2021. We geolocated users in 28 different countries and reconstructed a retweet network and cosharing network for each country. We identified communities of users exposed to no-vax content by detecting communities in the retweet network via hierarchical clustering and manual annotation. We collected a list of low-credibility domains and quantified the interactions and misinformation flows among no-vax communities of different countries.

**Results:**

The findings showed that during the pandemic, no-vax communities became more central in the country-specific debates and their cross-border connections strengthened, revealing a global Twitter antivaccination network. US users are central in this network, whereas Russian users also became net exporters of misinformation during vaccination rollout. Interestingly, we found that Twitter’s content moderation efforts, in particular the suspension of users following the January 6 US Capitol attack, had a worldwide impact in reducing the spread of misinformation about vaccines.

**Conclusions:**

These findings may help public health institutions and social media platforms mitigate the spread of health-related, low-credibility information by revealing vulnerable web-based communities.

## Introduction

### Background

The COVID-19 pandemic has extended vaccination from the purview of parents and health-compromised individuals to the purview of the broader public. Restrictions around vaccination created an additional potential to impact one’s personal freedom and the world economy, as well as one’s health. However, vaccination hesitancy continues to limit the impact of this highly effective intervention [1]: hundreds of thousands of lives were lost to COVID-19 that could have been prevented with vaccinations in the United States alone [2].

Vaccination hesitancy is a complex issue that has been associated with science denial [3], alternative health practices [4], and belief in conspiracy theories [5]. Among the many factors contributing to vaccine hesitancy is the spread of misinformation, especially on the web [6,7]. The impact of antivaccination content on online social media (OSM) may be compounded by the so-called echo chamber effect [8], in which users’ beliefs are reinforced through interactions with like-minded peers [9-11]. Created by the interplay of (1) homophily between users’ interactions and (2) polarization of the debate, echo chambers arise from a combination of the psychological tendencies of confirmation bias and selective exposure [12-14] together with algorithmic optimization for greater engagement at the cost of content diversity [15]. Importantly, echo chambers have also been found on OSM in the discussions around vaccination [16-19].

Thus far, scientific studies of the debate around vaccination on OSM have focused on specific countries [17,18,20,21] or English-speaking users [19]. Nevertheless, the COVID-19 pandemic has brought the vaccination discourse to a global scale [22], creating a deluge of international news around the development and deployment of COVID-19 vaccines, including low-quality content and misinformation [23]. The danger of this “infodemic” was acknowledged in mid-2020 by the United Nations and World Health Organization, which called for the member states to develop and implement the necessary action plans [1,24]. Thus, it is imperative to understand the flow of antivaccine—or no-vax—information not only nationally but also internationally to obtain a bird’s-eye view on the topic and inform effective communication campaigns.

To address this need, in this work, we focused on the Twitter (Twitter, Inc) platform by leveraging 316 million tweets related to vaccines in 18 different languages from a pre–COVID-19 pandemic era to April 2021 to quantify misinformation flows among users in no-vax communities across national borders and identify which countries are central in the global vaccination debate. To this end, we first investigated (1) how polarized, in terms of echo chambers phenomenon, the vaccination debate is in different countries over time to identify users in no-vax communities and (2) how susceptible, in terms of circulation of information, these no-vax communities are to low-quality information. We proposed a flexible, language-neutral community detection approach and combined it with human-in-the-loop expert knowledge to track polarization and echo chambers in different countries and time periods. We show that communities in which no-vax content was shared (1) increased in number during the pandemic, (2) became less isolated in the national vaccination debate, and (3) displayed much stronger cross-border connections than the rest of the users. Alarmingly, users in these communities tend to heavily rely on low-credibility information sources and to spread it across national borders, resulting in international spillovers of misinformation through a global no-vax network.

### Related Works

Vaccination deliberation on Twitter has been studied mainly in English and in the United States [25-27]. However, recently, the platform has gained attention from researchers also focusing on European countries. Before the pandemic, an analysis of the Dutch Twitter revealed an antagonistic relationship between an “anti-establishment” community and the community of journalists and writers, reinforcing the “arrogance of the elite” world view in the former [28]. On the Italian Twitter, the debate around vaccination revealed polarization in terms of retweets (RTs), where vaccine skeptics often mentioned vaccine advocates (generally in attacks), whereas the advocates seemed to ignore the skeptics altogether [17]. Outside Europe, a randomized study on Indonesian Twitter showed the importance of celebrity endorsement in message engagement and that the inclusion of the information source is associated with decreased propagation [29]. The COVID-19 pandemic has spurred increased attention to this topic. A recent examination of vaccine-critical actors on Francophone Twitter found that their place in discussions on vaccines has remained relatively constant during the pandemic compared with the mainstream media [20]. Furthermore, Crupi et al [18] studied the Italian Twitter during the rollout of the COVID-19 vaccinations, showing greater engagement across vaccine-supporting and hesitant communities in terms of mentions and similarity between the communities in the topics discussed.

Attempts to study the flows of vaccination discussion across borders have thus far been limited to dyadic relationships and English. A study of Canadian Twitter users found that most misinformation circulating on Twitter that was shared by Canadian accounts was retweeted from US-based accounts and that increased exposure to US-based information on Twitter is associated with an increased likelihood to post misinformation [30]. Beyond Twitter, Ng et al [22] examined news articles about COVID-19 from 20 countries, identifying the shift in narratives as the pandemic occurred. However, the data were limited to the English language and failed to capture the local language coverage. Unlike the previous studies, our study tracked the vaccination debate in the native languages of numerous countries to systematically study the flow of information (and potential misinformation) across national borders.

The most concerning aspect of the vaccination debates studied here is that misinformation may damage the confidence in the procedure. Controlled exposure studies have shown that web-based misinformation—especially misinformation that sounds scientific—negatively impacts vaccination intent in participants in the United States, United Kingdom [31], and New Zealand [32]. A panel study of US Twitter users found that the risk of average users occasionally sharing misinformation was alarmingly high, despite social bots’ contribution to misinformation sharing being “surprisingly low” [33]. Although some efforts have been made toward using high-quality, manually annotated data sets for identifying misinformation [34], the quality of the cited URL domains is often used as a gauge of the quality of the tweet’s content [35,36]. In this study, we used a similar approach by combining lists of low-credibility domains from several languages and countries.

Beyond content analysis, an important aspect of information and misinformation spread is the network structure underlying such dynamic processes. Echo chambers in the Twitter debate around the impeachment of former Brazilian President Dilma Rousseff have been shown to alter the diffusion of information between the supporters and opponents of the impeachment [37]. A similar methodology has been used to compare different topics across social media [8], highlighting that Facebook (Meta Platforms, Inc) shows a higher segregation of news consumption than Reddit (Reddit Inc). Along the same research line, the Random Walk Controversy (RWC) score [38] quantifies how controversial the topics discussed over a certain social network are as the probability of an average user being exposed to information from their own side versus from the opposing side. Although several studies address the presence of echo chambers on social media and their effect on information diffusion, little to no efforts have been devoted to understanding the echo chamber effects within cross-border information spreading, which we examined in this study.

## Methods

### Overview

**Figure 1:**
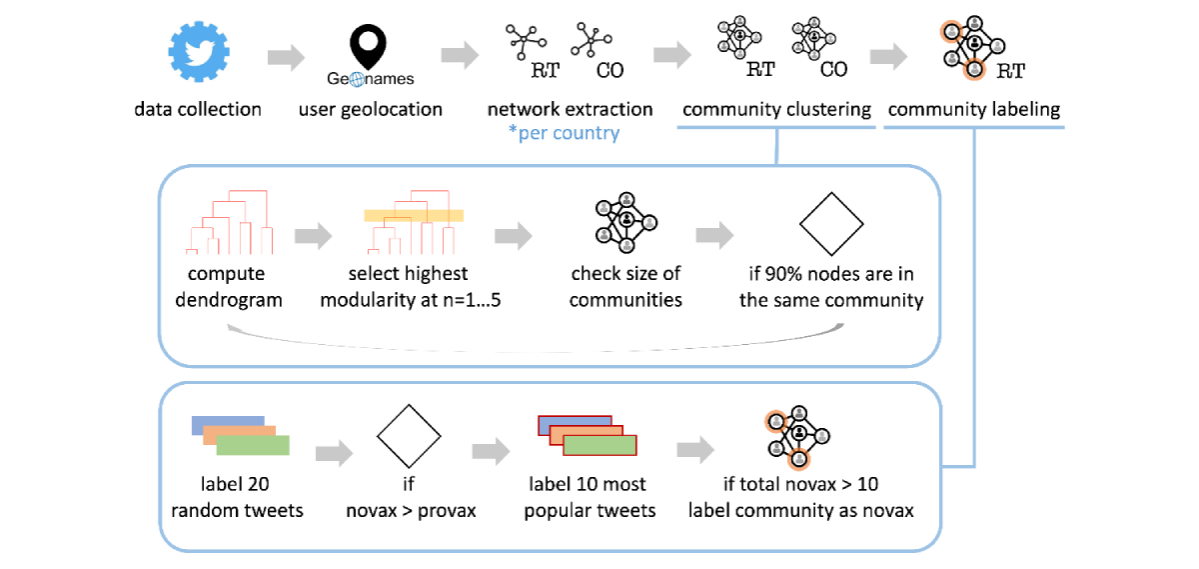
Flowchart of data processing, network extraction, community clustering, and community labeling. RT: retweet; CO: cosharing; novax: number of tweets labelled as discrediting vaccines per community; provax: number of tweets labelled as supporting vaccines per community.

The methodology of the data processing pipeline is outlined in the flowchart in Figure 1. First, we used the Twitter Streaming application programming interface (API) to collect a multilingual data set, which we geolocated using the GeoNames database [39]. To identify potential misinformation, we found lists of low-credibility domains in different languages. For the selected countries, we built 2 networks, RT and cosharing (CO; identified by users sharing the same URLs), and applied clustering to find communities. We then manually labeled (in 2 stages) samples of tweets from these communities to identify communities in which users were likely to encounter no-vax content. Finally, we computed several measures to quantify network polarization and information CO, as well as the intensity of cross-national interactions among no-vax communities.

### Data Set

**Figure 2:**
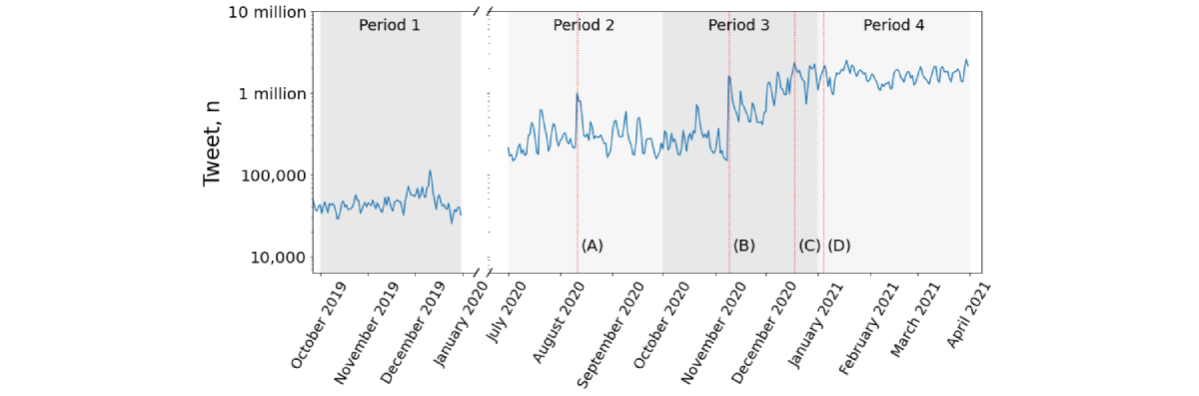
Volume of the vaccination debate on Twitter. Some external events with a substantial impact: (A) August 11, 2020: Sputnik V vaccine announced; (B) November 9, 2020: Pfizer-BioNTech vaccine announced; (C) December 18, 2020: Moderna vaccine announced; and (D) January 4, 2021: first AstraZeneca vaccine inoculation.

We began by assembling a list of vaccine-related words translated into 18 different languages (vaccine, novax, measles, MMR, vaccinated, etc), obtaining a set of 459 keywords (see queries here [40]). An existing list from previous work [17] was expanded by iterative querying of Twitter and expanding the list until no new keywords could be found. Native speakers were then recruited to translate the words into other languages and were instructed to include different common grammatical variations or local relevant keywords. For each language, we query the Twitter Streaming API [41] for the tweets containing the keywords in that language (translated by volunteer native speakers) and keywords in English by applying a language filter. For analysis, we chose four 3-month periods: (1) pre–COVID-19 pandemic period, from October 1, 2019, to December 31, 2019; (2) prevaccine period, from July 1, 2020, to September 30, 2020; (3) vaccine development period, from October 1, 2020, to December 31, 2020; and (4) vaccine rollout period, from January 1, 2021, to March 31, 2021. Figure 2 presents a summary of the daily volume of the data set. The volume increased by 2 orders of magnitude during the pandemic, from 6 million tweets in the 3-month pre–COVID-19 pandemic period to 39 million tweets in the prevaccine period to 91 million tweets in the vaccine development period to 178 million tweets in the vaccine rollout period. To check the completeness of our data, we ran an Historical API [42] in the pre–COVID-19 pandemic period with the same keywords. Owing to account suspension or post removal by the users themselves, a wide fraction of the tweets (72%) was not retrieved by this API, showing that such a data set cannot be retrieved by a retrospective search. Moreover, we took advantage of the passage of time to revisit the most notable accounts (present in the networks described subsequently) using the Twitter Get User API call [43] to check on their status, specifically noting whether the accounts have been suspended by the platform or deleted by the users.

### Geolocation

To capture country-specific dynamics of the social networks, we geolocated the users: we matched the location they provided in their description with the geographical database of locations from GeoNames. Manually verifying the matching accuracy, we filtered out >500 words often associated with nonlocations in this field. To further limit incorrect geolocations, we (1) removed the geolocation of users who changed their country locations during the observed period and (2) manually inspected users responsible for >50% of RTs between 2 pairs of countries in 1 period, assuming that a user who is heavily retweeted from another country is more likely to be wrongly geolocated. Under these conditions, we geolocated 48.7% of the users. This then allowed us to select countries for the study (as the focus was on the Western languages, we selected countries from Europe, North America, South America, and Oceania). To this end, we filtered countries with >2000 unique users in each period, obtaining 28 countries spanning 11 languages. Figure S1 in Multimedia Appendix 1 provides further details on the volume of tweets per language. The total number of different users geolocated in the chosen countries is 14.9 million, corresponding to 39.4% of the total number of users of the data set.

### Low-Credibility Domains

Following the previous literature on misinformation tracking [44,45], we collected a list of low-credibility domains. As sources of low-credibility websites, we relied on Bufale (Italian) [46], Wikipedia (English) [47], Media Bias/Fact-Check (English) [48], Le Monde (French) [49], and dwrean (Greek) [50], obtaining a list of 1732 domains. The fact that we were unable to find lists for less-used languages is an important limitation of this work, which we discuss in the *Discussion* section.

### Network Reconstruction

For each country, for each period, we built an RT network and a CO network. To limit the number of geolocation mismatches and filter users belonging to debates in other countries, we constrained the tweets considered for each country to the most common language in our data from that country among the languages spoken in the country (according to Wikipedia). The RT network is a directed weighted graph, where each node is a user, and the weight of the directed link *ij* is the number of times that user *i* retweeted user *j*. The CO network is an undirected weighted graph, where each node represents a user, and the weight of the undirected link *ij* is the number of unique URLs shared by both the users. In order to alleviate the computational cost of the network analyses, we filtered out the edges with weight equal to 1 (just 1 retweet) for the networks with more than 200.000 nodes. This filter affects the RT and CO networks in the United States (all periods), Brazil (PD, VD, and VR periods), Great Britain (VD and VR), and Spain and Mexico (VR period). This filter affects only the country-specific analyses of the RT networks, without influencing the later cross-country analysis and the findings about suspended accounts. When considering the constructed networks, we focused on the Giant Connected Component (GCC). On average, the GCC of the RT network contains 92% of its nodes, while the GCC of the CO network contains 76% of its nodes. In addition, we measured the Overlap Coefficient (OC) between the sets of users in the RT and CO networks. The OC is defined as the ratio of the size of the intersection of 2 sets, A and B, to the size of the smaller set, that is, OC(A,B) = |A ∩ B| / min(|A|, |B|). During the vaccine rollout period, the OC between the sets of users in the RT and CO networks increases from 0.72 (pre–COVID-19 pandemic period) to 0.86, indicating that more people are sharing URLs. The total number of users in the reconstructed RT and CO networks is 2.7 million.

### Hierarchical Clustering

Next, we applied a community detection algorithm to cluster the users of the RT and CO social networks. Because the goal was to find a small number of large groups of users, we adopted hierarchical clustering, instead of unsupervised algorithms (eg, Louvain), which finds the optimal partitioning with a very large number of often small communities. We used Paris, an agglomerative hierarchical clustering algorithm induced by the probability of sampling node pairs [51]. Next, we cut the dendrogram to have a reasonable number of communities that are not too unbalanced following the steps listed here:

1. Build the dendrogram of the hierarchical clustering.

2. Compare the partitions obtained with cutoffs at heights 2, 3, 4, and 5 (ie, having this number of communities).

3. Pick the partition with the highest modularity.

4. If >90% of the nodes are in the same community, compare the partitions with cutoffs at the following 5 heights and repeat from step 3.

Using this procedure, we ensured that 90% of the users were partitioned into at least 2 communities but no >5 communities (although it is possible to have many small communities that comprise <10% of the nodes). Using this method, we found, on average, 6.7 communities in the RT networks and 5.0 communities in the CO networks, with a maximum of 20.

### Labeling

To identify communities in which users were exposed to no-vax content, we labeled a sample of tweets shared in each community. First, we filtered out small communities by considering only those with >1% of the users of the network, resulting in 400 communities. Next, we randomly sampled 20 tweets from each community, resulting in a total of 8000 tweets. A total of 12 people were involved in labeling, all of whom had a background in vaccine debate and knowledge of the language used in the tweet to label. Furthermore, we translated all tweets into English using Google Translate to allow for cross-checking. Each person labeled between 600 and 1000 tweets, with an overlap of 20 tweets with other annotators. The tweets were labeled as “pro-vax,” “no-vax,” or “other.” We labeled tweets as pro- or no-vax only if they were clearly supporting or discrediting vaccines, respectively. Therefore, more than half of the labels were “other,” comprising nonrelevant posts, posts with unclear positions, discussions on other policies, and all generic pieces of news that did not express a stance. The task of distinguishing between pro vaccination and antivaccination stances proved to be fairly easy, with Cohen κ computed on an overlapping set at κ=0.84 (only 3% received different labels). By contrast, the task involving the “other” label proved to be more difficult, with κ=0.51 for the 3-class setting (disagreement of 26%), mainly because of the confusion between “other” and “pro-vax” labels (disagreement of 20%). However, note that we were only interested in distinguishing between the antivaccination stance and the rest.

To improve the quality of the labels, we then proceeded to a second round of annotation, focusing on the communities that have a majority of content with a no-vax stance. Specifically, we chose the communities with a majority of no-vax tweets and annotated the 10 most popular tweets in each (excluding the 50 most popular tweets in the whole network). The second labeling stage encompassed 82 communities, totaling in 820 tweets. At this stage, the Cohen κ for the 3 classes was 0.64. Finally, we defined a community as no-vax if the total number of “no-vax” labels in the rounds was >10, resulting in 58 communities. Because some networks had >1 antivax community, we had 52 networks with a no-vax community, that is, a community where users were substantially exposed to no-vax content (Figure S2 in Multimedia Appendix 1).

### Clustering Robustness

Next, we assessed the robustness of our approach to determine if our methodology influenced the results. To do so, we compared the communities previously identified in the RT networks with those obtained using 2 alternative partitioning algorithms: Louvain and Paris hierarchical clustering with a cutoff of 10. We chose the Louvain algorithm for its popularity in community detection problems in social network analysis and Paris with a cutoff of 10 for comparing the results obtained with a different parameter in the cutoff of the same dendrogram. We quantified the number of labeled tweets shared by users in the new clustering and categorized communities as “no-vax” using 2 different thresholds: the “majority threshold” and the “strict threshold.” The former was applied when “no-vax” labels outnumbered “pro-vax” labels, while the latter was used when “no-vax” labels surpassed both “pro-vax” and “other” labels. This yielded 4 alternatives to our method: Louvain partitioning with majority threshold, Louvain partitioning with strict threshold, hierarchical clustering with majority threshold, and hierarchical clustering with strict threshold.

To evaluate the robustness of our methodology, we calculated the accuracy for each network as the proportion of users classified in the same group as the previous method (either “no-vax” or “not no-vax”). Our results demonstrate a high level of robustness, with average accuracies of 0.90 and 0.94 using Louvain partitioning with majority and strict thresholds (SD 0.15 and 0.10) and 0.92 and 0.95 using hierarchical clustering (SD 0.15 and 0.10), respectively. These findings support the consistency of the results presented in this paper, which are not overly dependent on the methodology used to detect and label communities.

### RWC Score

Following previous literature [10,17,52], we used the RWC score to quantify the polarization between the communities labeled as no-vax and the rest of the network. Given an RT network, partitioned into 2 clusters X and Y, RWC is calculated as P_XX_ P_YY_−P_XY_ P_YX_, where P_XY_=P (a random walk ended in Y started in X). Intuitively, it represents the difference in probability for an average user in the network to be exposed to information from their own side versus that from the opposing side. Spanning (0, 1), an RWC close to 1 represents a polarized social network with 2 distinct groups that do not endorse each other’s opinions, whereas an RWC close to 0 represents a noncontroversial topic where both opinions are equally likely to be received.

### Normalized Mutual Information

We quantified the echo chamber effect by measuring the extent to which users from different RT communities shared the same sources of information (as quantified by the CO network), as a proxy for the information siloing in an echo chamber. To do this, we used normalized mutual information (NMI) [53] to gauge the similarity between the RT and CO communities obtained by hierarchical clustering, using the normalized_mutual_info_score module in the Python package *scikit-learn*. Spanning (0, 1), an NMI of 1 means that the community structure is the same between the networks, whereas a low NMI indicates different community structures. Note that this metric did not use the opinion leaning determined in the labeling step and thus could be computed for any country/period network, regardless of whether it had a no-vax community.

### Normalized RT Volume

To assess the extent to which one country retweets another, we computed a normalized retweeting volume for each pair of countries. To this aim, we started by the total number of RTs from country *i* to country *j* (which we have indicated as *a_ij_*). Then, we divided it by the total number of RTs by users in country *i* (indicated as *s_i_*^out^) and the total number of RTs to users in country *j* (indicated as *s_j_*^in^), and we multiplied it by the total number of RTs by all countries (indicated by W). The normalized retweet volume *n_i_*_j_ is thus equal to the following:



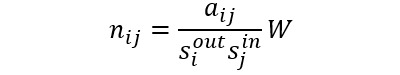



Note that 
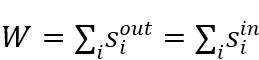
 , and 
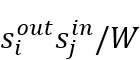
 is the expected number of RTs from country *i* to country *j* in the random graph with the same node strengths. Hence, *n_ij_* >1 if *i* retweets *j* more than it would in a random baseline context. As the vast majority of RTs were within the same countries, 
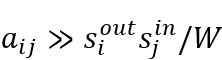
 if 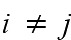, otherwise 
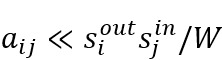
. To focus on cross-border interactions, we consider *n_ii_* = 0, for any *i*.

### Cross-Border Interactions Between No-Vax Communities

We measured the strength of ties among the users in no-vax communities in different countries by comparing the number of RTs among these users with the number of RTs among the rest of the users in the same countries. In particular, we define *V_i_^K^* as the set of users in communities with stance *K* in a country *i*, where *K* can be *A* (antivax) or *O* (others). We define *W_ij_^K^* as the number of RTs from users in *V_i_^K^* to users in country *j* with the same stance *K*, *V_j_^K^*. Thus, one can measure the density, 
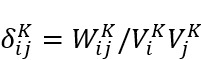
 the ratio of observed RTs; and the total possible pairs between sets *V_i_^K^* and *V_j_^K^*, that is, the probability that 2 random users in *V_i_^K^* and *V_j_^K^*, respectively, are connected. For each pair of countries, we analyzed if 
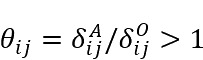
, it means that the probability that 2 random users in no-vax communities in countries *i* and *j* are connected is higher than the probability that 2 random users in the rest of the network of the same countries are connected.

### Ethical Considerations

Although the data were collected using the platform’s own API, resulting only in posts that were posted publicly, it is possible that some users were unaware of the scope of their potential audience. Thus, we follow the platform’s terms of service and share only the IDs of the tweets so that when the data are recollected, deleted content will not be available (notably limiting the reproducibility of any Twitter-based study). Thus, the data that are shared do not contain any identifiable information about the poster or any other information except the numeric ID of the tweet, preserving the privacy of the user to the extent that they choose to keep their posts public on the platform. This will affect the reproducibility of the study, as some content may be deleted by the users over time. Furthermore, the multinational nature of the data captured wildly varying biases in the way people around the world are able to access the internet or Twitter specifically. Local barriers to access to the internet and local blocks of the platform itself shape the communities captured in this study. For instance, dissidents or those who wished to remain anonymous would likely not have shared their location information on their profile and would not have been captured as being a part of a country’s discussion. The construction of retweet and cosharing networks also necessitates enough activity by the user to be included in the analysis, biasing our results to those who are more active in the conversation, especially in retweeting and sharing URLs. Moreover, the data may have captured vulnerable groups, including those who experienced or who were at risk of specific health conditions, those who had financial barriers to health care, and even those who were more susceptible to misinformation. Despite the negative connotation around “no-vax” communities, users found to propagate harmful information may first and foremost endanger themselves by following faulty advice. Thus, we would discourage the future researchers from publishing verbatim tweet text to preserve user privacy.

Finally, in this paper, we present tools that may be used to track and profile groups of Twitter users around a topic. These tools may then be used by both the platform and the government. However, such tools may also be used to target communities for harassment, doxing (providing private user information to harm or intimidate the person), and other abuses. On the one hand, it is the responsibility of the platforms and their communities to uphold the civil code of conduct and block the abusers. On the other hand, we call for the research community, as well as corporate and governmental actors, to use these tools ethically, with minimal harm to the participants.

## Results

### Polarization of the Vaccination Debate

**Figure 3:**
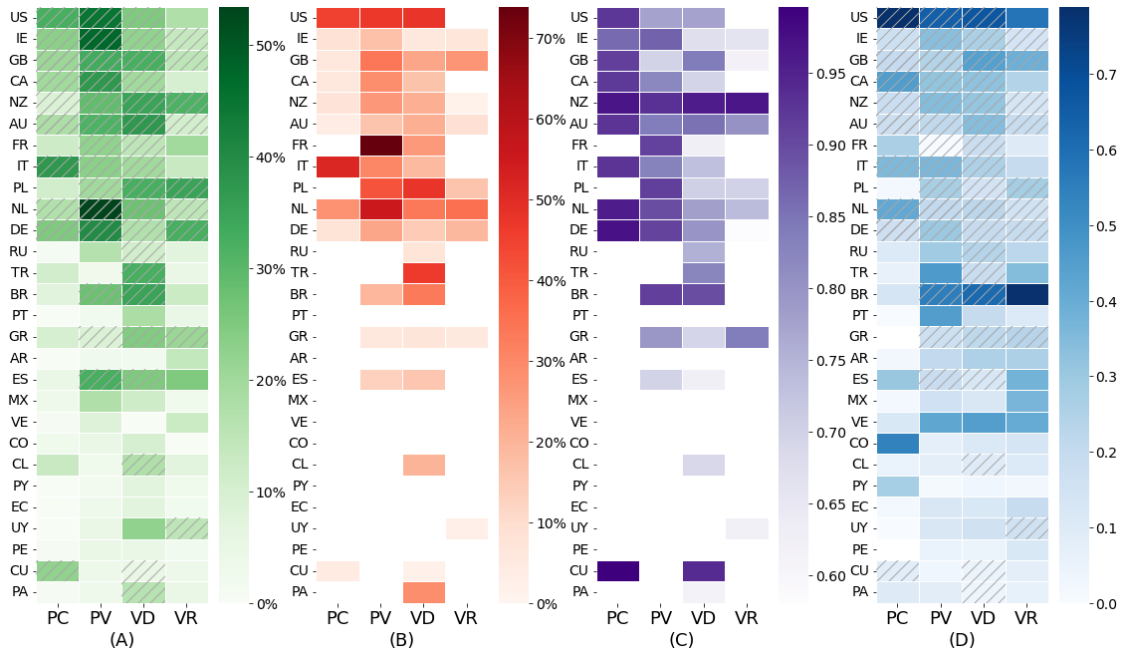
Characterization of no-vax communities for each country and period considered via retweet (RT) and cosharing (CO) networks. (A) Proportion of tweets labeled as no-vax. (B) Proportion of users in no-vax communities with respect to the size of the RT networks (average in the 4 periods: 16.9% [SD 0.18], 30.9% [SD 0.18], 23.1% [SD 0.14], and 13.7% [SD 0.12]). (C) Random Walk Controversy between no-vax communities and the rest of the networks (average in the 4 periods: 0.94 [SD 0.04], 0.84 [SD 0.08], 0.76 [SD 0.10], and 0.73 [SD 0.12]). (D) Normalized mutual information between RT and CO communities. Countries with no-vax communities are marked with dashed lines. PC: pre–COVID-19 period; PV: prevaccine period; VD: vaccine development period; VR: vaccine rollout period. AR: Argentina; AU: Australia; BR: Brazil; CA: Canada; CL: Chile; CO: Colombia; CU: Cuba; DE: Germany; EC: Ecuador; ES: Spain; FR: France; GB: Great Britain; GR: Greece; IE: Ireland; IT: Italy; MX: Mexico; NL: Netherlands; NZ: New Zealand; PA: Panama; PE: Peru; PL: Poland; PT: Portugal; PY: Paraguay; RU: Russia; TR: Turkey; US: United States; UY: Uruguay; VE: Venezuela.

We began by examining different measures of polarization and no-vax activity in different countries over the 4 periods. Figure 3A shows that a high presence of no-vax tweets in a certain country and period is often associated with the presence of a community labeled as no-vax (dashed lines). This implies that no-vax content is generally clustered and not homogeneously distributed in the RT network, suggesting that the debate is polarized, as illustrated subsequently. Furthermore, we found that no-vax communities were generally present in the English-speaking countries (eg, compared with the Spanish-speaking ones). However, some of the relatively largest country-specific no-vax communities appeared in France, Italy, the Netherlands, Poland, and the United States (Figure 3B). No-vax communities were particularly present in the prevaccine and vaccine development periods, where they also spanned a larger fraction of users compared with the other periods. Turning to potential echo chambers in these networks, we found that the RWC score was overall very high (Figure 3C), indicating that the vaccination debate was generally highly polarized. However, it decreased substantially over time, suggesting that the users in no-vax communities became less isolated in the vaccination discourse during the COVID-19 pandemic. Furthermore, we investigated whether the users in the no-vax communities were exposed to information sources that were different from those that the rest of the users were exposed to by considering NMI. Despite the NMI being independent of the labeling of the communities, Figure 3D shows that, on average, the NMI of the networks with a no-vax community was higher than that of the others (0.27 vs 0.22, *P*<.05), indicating that the users in no-vax communities tended to have common information sources. Some countries, such as the United States and Brazil, showed an especially high NMI, indicating that the polarization in the RT network was reflected in the different content shared. The Spanish-speaking countries, conversely, were less polarized than the English-speaking countries (average 0.15 vs 0.33, *P*<.001).

### Characterizing the Users in No-Vax Communities

**Figure 4:**
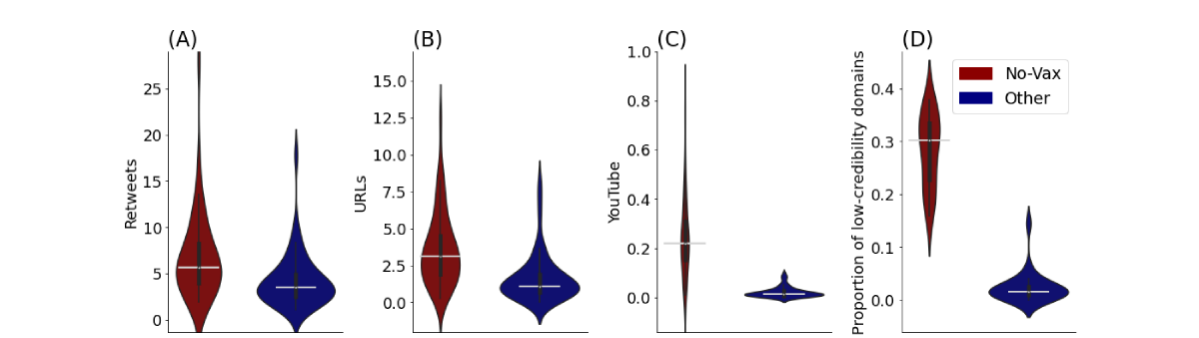
Behavior of users in no-vax communities versus those of other users. (A) Average retweets, (B) average URLs, (C) average YouTube URLs, and (D) proportion of low-credibility domains shared by users. Note that low-credibility domains were collected only in Italian, French, English, and Greek; therefore, the plots refer to countries speaking these languages. No-Vax: discrediting vaccines. Other: non-discrediting vaccines.

Considering the behavior of the users in no-vax communities, we found that they were more likely to retweet (Figure 4A) and share URLs (Figure 4B), especially URLs to YouTube (Figure 4C), than the other users. Furthermore, the URLs they posted were much more likely to have been from low-credibility domains (Figure 4D) compared with those posted in the rest of the networks. The difference is remarkable: 26% of the domains shared by no-vax communities came from lists of known low-credibility domains versus only 2.4% of those cited by the other users came from lists of known low-credibility domains (*P*<.001). The most common low-credibility websites among the no-vax communities were *Zero Hedge*, *LifeSiteNews*, *Daily Mail* (considered right-biased and questionably sourced), and *Children's Health Defense* (conspiracy/pseudoscience). These findings extend the existing literature on English-language vaccination rhetoric to a multilingual, international scope by confirming the elevated social engagement in antivaccination communities [54] and provide additional evidence of the misleading nature of the popular COVID-19 pandemic–related YouTube videos [55].

**Figure 5:**
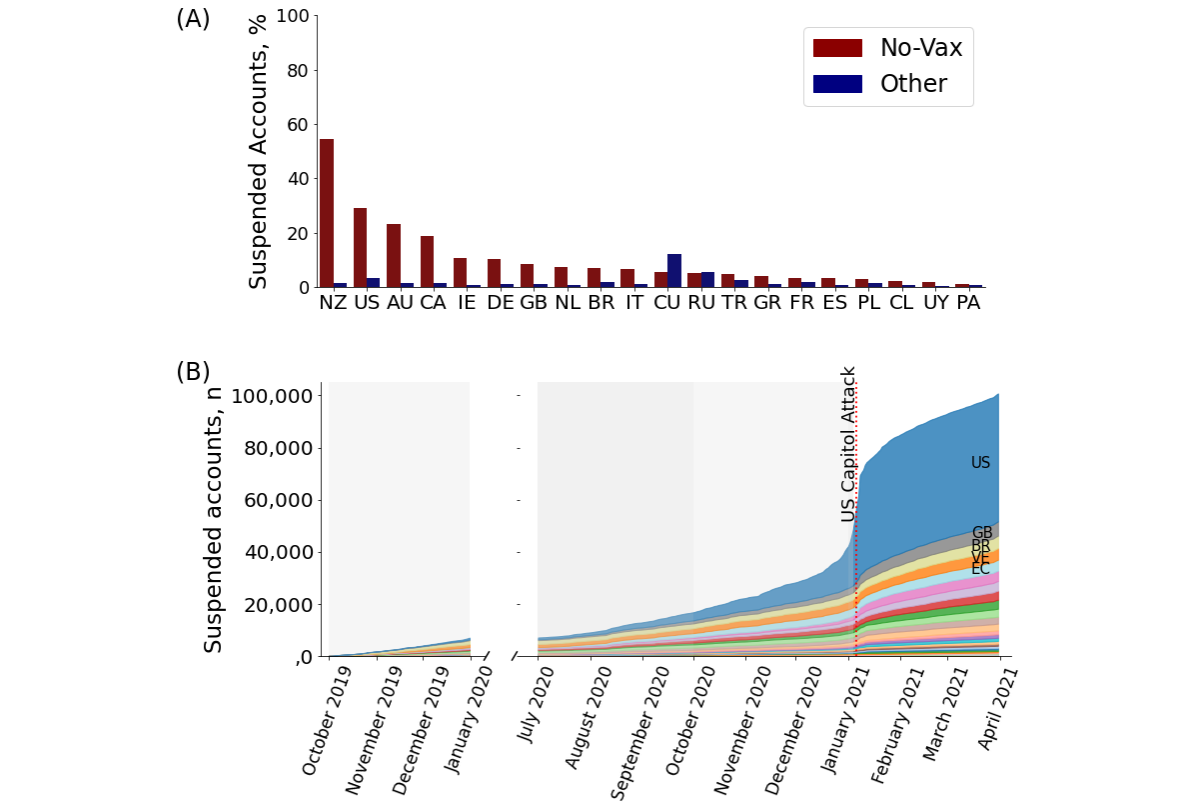
Suspended users per country in no-vax communities. (A) Average proportion of suspended accounts per country in the period in which no-vax community has been detected, computed separately for no-vax side and rest of users. (B) Number of suspended accounts as a function of the date they posted their last tweet, colored by country. No-Vax: discrediting vaccines. Other: non-discrediting vaccines. AR: Argentina; AU: Australia; BR: Brazil; CA: Canada; CL: Chile; CO: Colombia; CU: Cuba; DE: Germany; EC: Ecuador; ES: Spain; FR: France; GB: Great Britain; GR: Greece; IE: Ireland; IT: Italy; MX: Mexico; NL: Netherlands; NZ: New Zealand; PA: Panama; PE: Peru; PL: Poland; PT: Portugal; PY: Paraguay; RU: Russia; TR: Turkey; US: United States; UY: Uruguay; VE: Venezuela. No-Vax: plots refer to users in no-vax communities. Other: plots refer to users in other communities.

Next, we investigated the effects of content moderation by Twitter on the vaccination debate. We found that the average proportion of suspended accounts in no-vax communities was much larger than that among the rest of the users for each country and period considered (average 13.3% vs 1.8%, *P*<.001; Figure 5A). The highest proportions of suspended accounts were found in the English-speaking countries, Germany, and the Netherlands, which also showed a larger presence of no-vax content, than in the other countries. Furthermore, a large portion of suspensions came after the January 2021 US Capitol attack in Washington, DC [56] (Figure 5B). The proportion of suspended accounts from the United States increased from 38% before January 1 to 77% during the days around the Washington riots (January 1-12). Note that (1) 89% of the US users who were suspended belonged to the no-vax community in the vaccine development period; (2) the account realDonaldTrump (suspended on January 8) was one of the most popular accounts among the no-vax communities of the first 3 periods; and (3) in the last period, a no-vax community was not present in the US RT network, indicating that the suspension of US accounts following the Washington riots heavily impacted the vaccination debate on Twitter. These findings suggest that political leaning is often associated with strong stances taken in the vaccination debate (in line with previous literature [17,21]) and that actions taken in the political domain may greatly impact the quality of the public health discourse.

### Cross-Border Information Spillover in the Global Vaccination Debate

**Figure 6:**
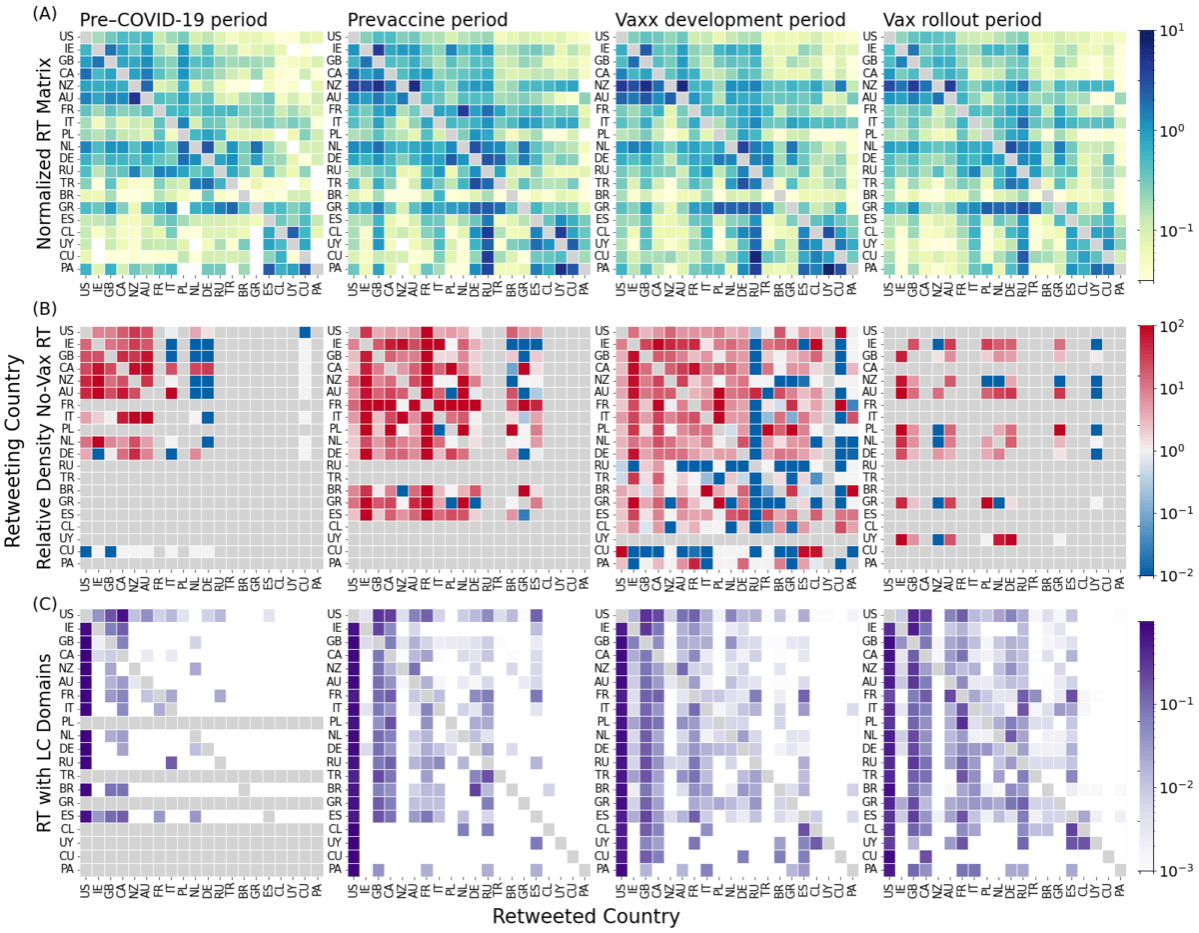
Cross-border information flows in the global vaccination debate. (A) Normalized retweet (RT) matrix. Normalized number of retweets (excluding diagonal elements, colored in gray). (B) The ratio between probability of interactions between users in no-vax communities and rest of the networks from the same pair of countries (see the Methods section). Darker red (blue) elements of the matrices represent higher (lower) tendency of cross-border interactions between users in no-vax communities with respect to other users. Countries without no-vax communities are colored in gray. (C) Proportion of URLs that come from the retweeted country among the low-credibility (LC) domains imported from the retweeting country. Countries importing ≤10 low-credibility URLs are colored in gray. Diagonal elements are also gray, as our focus was on cross-country interactions. Element aij of each matrix represents the information flow from country j to country i. In the plots, only the countries with at least 1 no-vax community in the 4 periods are represented. The extended version is Figure S4 in Multimedia Appendix 1.

Next, we quantified the information spillover across countries by considering the number of RTs from one country to another, normalized by the total number of RTs produced and received in the 2 countries (see Normalized RT Volume in the Methods section; Figure 6A). First, one can observe language homophily, indicated by the darker regions in the top left (English) and bottom right (Spanish) of the panels, as well as the pair Portugal-Brazil, in all periods. The darker patches corresponding to the interactions between Germany and the Netherlands and those between Germany and Turkey also reflect possible cultural or expat relationships. Second, the cross-border interaction matrices are not symmetrical: information generally flows in a preferred direction. For instance, the Spanish-speaking countries retweeted the English-speaking countries much more than the English-speaking countries retweeted the Spanish-speaking countries. Note that the United States is central in the global information flow, being a net exporter of information to the rest of the world when comparing inflows versus outflows of information for each country (Figure S3 in Multimedia Appendix 1). Interestingly, from the prevaccine period, Russia also became a net exporter, especially to South American countries (Figure S3 in Multimedia Appendix 1): some of the most used hashtags in the prevaccine and vax development periods are *#sputnikesesperanza* and *#sputnikparaelpueblo*.

In Figure 6B, we quantified the strength of the cross-border interactions among the users in no-vax communities compared with that among the rest of the users (see Cross-Border Interactions Between No-Vax Communities in the Methods section). We found that cross-border interactions among the users in no-vax communities were generally much stronger, sometimes by orders of magnitude, than the interactions among the rest of the users, creating a tightly knit global no-vax network. In particular, the users in the no-vax communities of the English-speaking countries, Germany, and the Netherlands were tightly connected in all periods. By contrast, the users in the no-vax communities from Cuba and Russia were isolated (adding to their unusual user suspension statistics). Again, cross-border interactions can be asymmetrical: for instance, in the pre–COVID-19 pandemic period, the users in the no-vax communities in Germany and the Netherlands retweeted the users in the other countries, but not vice versa.

Finally, we focused on misinformation flows across countries by considering the fraction of low-credibility domains imported per country (Figure 6C), that is, the fraction of tweets pointing to low-credibility URLs, over the total number of RTs from one country to another. We stress that we considered flows of low-credibility information across borders spread by both humans and bots, without engaging in the difficult task of distinguishing them, as we were interested in quantifying how exposed a certain country A is to misinformation coming from country B. As in the previous case, the matrices show a clear asymmetry. The US users were responsible for exporting a large fraction of misinformation to the rest of the world: 68% of all the low-credibility URLs retweeted worldwide came from the United States (average over the 4 periods), a proportion much higher than the total volume of URLs (42%) retweeted from the United States.

Interestingly, the fraction of low-credibility URLs from the United States dropped from 74% in the vax development period to 55% in the vax rollout period. This large decrease can be directly ascribed to Twitter’s moderation policy: 46% of cross-border RTs of US users linked to low-credibility websites in the vax development period came from accounts that were suspended following the US Capitol attack (Figure S5A in Multimedia Appendix 1). Note that Twitter’s account purge substantially impacted the misinformation spread worldwide: the proportion of low-credibility domains in the URLs retweeted from the United States dropped from 14% to 7%. Finally, despite not having a list of low-credibility domains in Russian, Russia was central in exporting potential misinformation in the vax rollout period, especially to Latin American countries. In these countries, the proportion of low-credibility URLs coming from Russia increased from 1% in the vax development period to 18% in the vax rollout periods (Figure S5B in Multimedia Appendix 1).

## Discussion

### Principal Findings

The international, multilingual nature of the data we present here supports the ongoing efforts in monitoring the non-English debate around the topic of vaccination [57-59]. Using this information, we reveal the increasingly globalized nature of the vaccination debate as the COVID-19 vaccines were proposed, developed, and deployed. This increased globalization had a marked impact on vaccine-hesitant discourse: not only did the prominence of the no-vax communities increase within individual countries but their cross-border connections also strengthened around the world. We showed that the users in these communities are much more prone to sharing potential misinformation than other users, even across national borders.

Furthermore, the real-time nature of the data collection allowed us to capture Twitter’s content moderation efforts, which proved to be uneven both across countries and time. The users blocked immediately following the January 6 Washington riots were responsible for a substantial amount of misinformation spread—both within the United States and, crucially, internationally. Thus, we paint a picture of a “global no-vax Twitter network” that calls for the international collaboration of both public health and technology experts.

### Limitations

Our study has several important limitations. First and foremost, it is well known that Twitter users are not a representative sample of the real population but are biased toward more educated, urban, younger, and male individuals [60]. Furthermore, Twitter use wildly differs among the countries under consideration, so cross-country comparisons should be taken with caution. With respect to this, the geolocation task also introduced some bias in the results due to the different fraction of missing accounts across countries. However, to the best of our knowledge, there are no means to collect real-time, representative data at this spatial and temporal scale. Note that we did not engage in bot detection, as this task is notoriously difficult [61], and, most importantly, misinformation can be spread by complex interactions by bots and humans [44]. Moreover, our study was limited to 11 chosen languages and to the 4 languages for which low-credibility domains were collected, a limitation necessary to control the cultural heterogeneity of the data analyzed. However, the fact that low-credibility domains in other languages were not found (for instance, it was challenging to find a reputable list of low- or high-credibility domains in Russian) means that the potential misinformation flows presented here are a lower bound—one which should be expanded using additional resources. Furthermore, the content considered for this study is limited to the keywords and the query processing of the Twitter search engine. Note that we did not check for spelling errors, which may lead to underdetection of some tweets. However, we did perform a relevance check on a random selection of 300 tweets, resulting in 7% of pet-related tweets, 6% of nonrelevant tweets, and the remaining tweets relevant to vaccination. As we did not modify the keywords as events unfolded—notably when new vaccines were developed—in the aim of keeping a consistent methodology allowing for comparison over time, some content pertaining to time-specific keywords was missed (although we were still able to capture a large amount of discussion around these developments with existing keywords).

Future work should also be devoted to including countries from Africa and Asia, as well as to update and extend the list of low-credibility information sources to other languages. For the latter task, one could leverage the identification of no-vax communities—more susceptible to share low-credibility information—proposed in this study. Other possible limitations of this study include the method used to identify no-vax communities, hierarchical clustering of the RT network and labeling of the popular tweets in the resulting communities, which may have been sensitive to the thresholds adopted. However, note that this method was not aimed at detecting the stance of single users about vaccination but at identifying large clusters of users exposed to a certain kind of antivaccine narrative.

### Broader Impact

Despite the platform’s tweet flagging and removal policies around COVID-19 [62,63], it is the bout of account suspensions around the Washington riots that made the most impact on the national and international spread of vaccine-related misinformation, suggesting that political concerns elicit much stronger curbing of the freedom of speech than health concerns. It is possible that the effects of this event changed the social media landscape itself, with platforms such as Truth Social appearing in the aftermath of the event. More documentation of the causal link between web-based misinformation and adverse health outcomes may provide a more solid ground for making censorship decisions for both the platforms and the politicians governing them. For instance, a randomized controlled trial in the United Kingdom and the United States showed that “relative to factual information, recent misinformation induced a decline in intent of 6.2 percentage points” [31].

Further, the Centers for Disease Control and Prevention and the Kaiser Family Foundation estimate that the lack of action early in the pandemic may have contributed to deaths of hundreds of thousands by June 2021 [2]. Furthermore, this study illustrates the impact of 1 social media platform’s editorial policies on the international public health discourse, especially when the country involved is as culturally influential as the United States. Without examining in detail the content shared by the suspended accounts, we cannot be certain that the accounts indeed were sharing harmful content. Monitoring the censorship activities of major platforms (triggered by either internal policies or governments’ requests) is important for assessing the users’ freedom of speech. For instance, the Electronic Frontier Foundation has recently criticized social media platforms for blocking political dissidents who a decade ago used the same platforms to “push for political change and social justice” [64]. Fortunately, “de-platforming as censorship” is a topic of ongoing deliberation at the European Union’s Internet Governance Forum involving civil society and government representatives [65].

An international perspective may also benefit the tracking of malicious actors, such as semiautomated or fully automated accounts, networks of colluding agents, and sources of poor-quality content. It has been shown that accounts identified as Russian trolls were more likely to tweet about vaccination before the pandemic [66]. During the pandemic, Russian trolls often posted misinformation concerning the personal dangers of vaccines, purported civil liberty violations, and vaccine conspiracies [67]. Since the beginning of the Ukraine war, it has been noted that antivaccine content has diminished dramatically, potentially because of the additional blocking of Twitter in Russia and refocusing of the conspiratorial attention on Ukraine [68]. Our findings suggest that changes in governance and censorship may encourage or discourage the flow of potential misinformation from states with known affinities.

## Appendix

Multimedia Appendix 1 Additional figures for the data selection, annotation and analysis.

## Abbreviations

API: application programming interface
CO: cosharing
NMI: normalized mutual information
OSM: online social media
RT: retweet
RWC: Random Walk Controversy

## Data Availability

All codes generated in the project have been deposited to GitHub [69]. All data collected in the project have been deposited to Zenodo [70].
