# Supplementary material for: Global Misinformation Spillovers in the Vaccination Debate Before and During the COVID-19 Pandemic: Multilingual Twitter Study
Source: JMIR Infodemiology. 2023 May 24;3:e44714. doi: 10.2196/44714 (PMC10226529; doi:10.2196/44714)
Supplement: Multimedia Appendix 1 [file infodemiology_v3i1e44714_app1.docx]

**Multimedia Appendix 1**

**Global Misinformation Spillovers in Vaccination Debate Before and During COVID-19 Pandemic: Multilingual Twitter Study**

Below, additional figures elaborate on the data selection, annotation, and analysis.


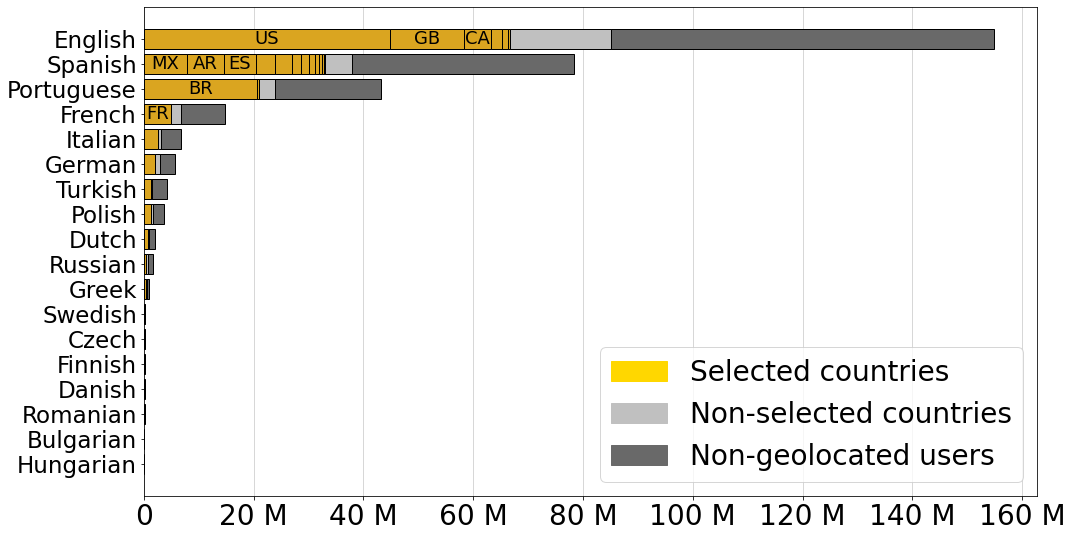


**Figure 1: Tweets volume per language and country.** Number of tweets in the data collection by language and geolocated country (only largest countries labeled).

The complete list of countries (in 2-letter ISO standard): US, BR, AR, GB, ES, MX, FR, CA, TR, VE, AU, CO, IT, CL, DE, PT, IE, PY, EC, RU, UY, NZ, PL, NL, PE, CU, PA, GR.


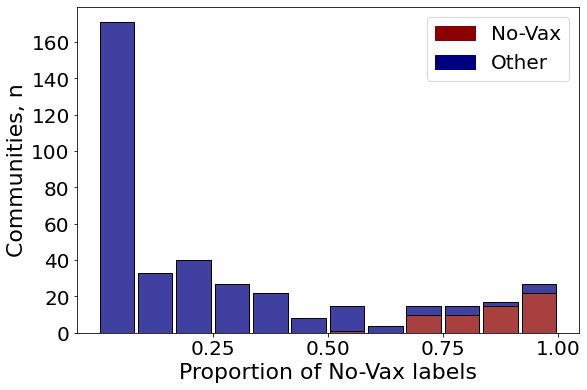


**Figure 2: Number of communities with a certain proportion of tweets labelled as no-vax in the first labelling.** In red communities with more than 10 anti-vax labels in the two rounds (no-vax communities). We studied 400 communities, and 58 of them were labeled no-vax.


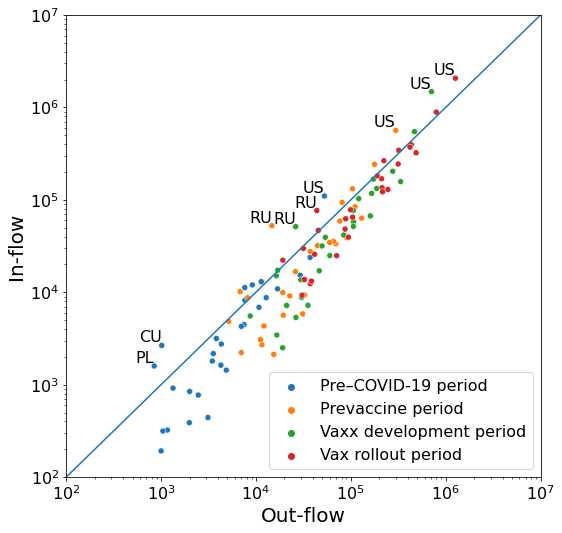


**Figure 3: Comparison between information inflows and outflows.** The scatter plot represents the comparison between the number of cross-country RTs from users in each country and period (out-flow) with the number of cross-country RTs to users in the same country and period (in-flow). We labeled the points with in-flow > 1.5 · out-f low. We observe that U.S. (all periods) and Russia (from pre-vaccine period) are the main information exporter, being retweeted much more than they retweet.


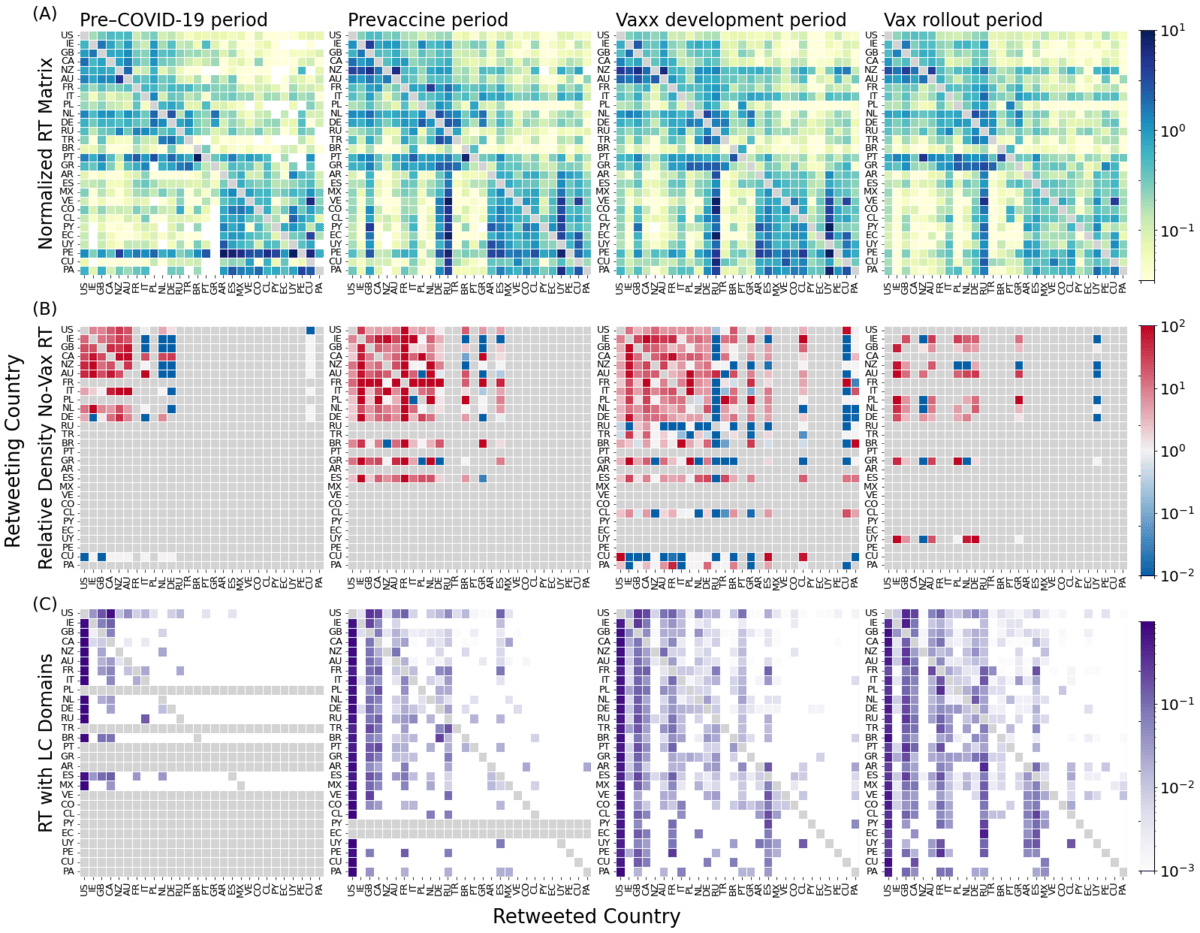


**Figure.4: Cross-border information flows in the global vaccination debate.** Extended version of Fig. 6.


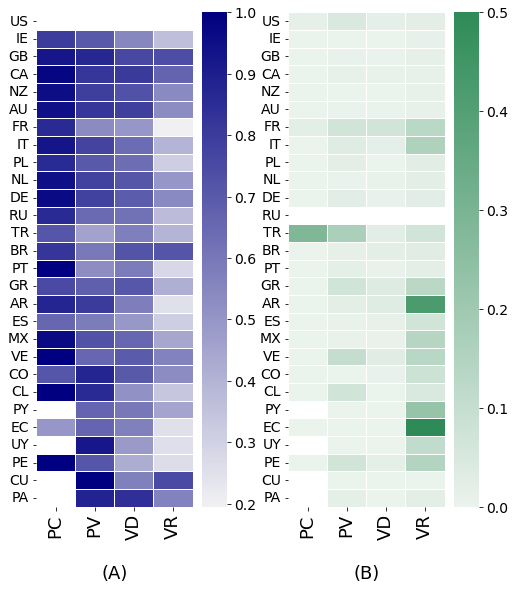


**Figure 5: URLs with low-credible domains exported by U.S. and Russia.** (a) Proportion of low-credible URLs imported from U.S., for each country and period considered. Overall, the proportion of URLs that comes from U.S. is 93%, 79%, 74%, 56%: this highlights the central role of U.S. in the spread of misinformation and the sharp decrease after the suspension of the accounts. (b) Proportion of URLs imported from Russia, for each country and period considered. In general, countries import 1%, 2%, 1%, 10% of the low-credible domains from Russia. The marked growth observed in the last period is particularly notable in Latin American countries, where the proportion of low-credible domains coming from Russia increase from 1% in VD period to 18% in VR period.
